# CIGuide: in situ augmented reality laser guidance

**DOI:** 10.1007/s11548-019-02066-1

**Published:** 2019-09-11

**Authors:** Zoltán Bárdosi, Christian Plattner, Yusuf Özbek, Thomas Hofmann, Srdjan Milosavljevic, Volker Schartinger, Wolfgang Freysinger

**Affiliations:** Medical University Innsbruck, Innsbruck, Austria

**Keywords:** Magnetic tracking, Laser guidance, Robotic control, Augmented reality, Optical tracking, Microscope, Navigated surgery

## Abstract

**Purpose:**

A robotic intraoperative laser guidance system with hybrid optic-magnetic tracking for skull base surgery is presented. It provides in situ augmented reality guidance for microscopic interventions at the lateral skull base with minimal mental and workload overhead on surgeons working without a monitor and dedicated pointing tools.

**Methods:**

Three components were developed: a registration tool (Rhinospider), a hybrid magneto-optic-tracked robotic feedback control scheme and a modified robotic end-effector. Rhinospider optimizes registration of patient and preoperative CT data by excluding user errors in fiducial localization with magnetic tracking. The hybrid controller uses an integrated microscope HD camera for robotic control with a guidance beam shining on a dual plate setup avoiding magnetic field distortions. A robotic needle insertion platform (iSYS Medizintechnik GmbH, Austria) was modified to position a laser beam with high precision in a surgical scene compatible to microscopic surgery.

**Results:**

System accuracy was evaluated quantitatively at various target positions on a phantom. The accuracy found is 1.2 mm ± 0.5 mm. Errors are primarily due to magnetic tracking. This application accuracy seems suitable for most surgical procedures in the lateral skull base. The system was evaluated quantitatively during a mastoidectomy of an anatomic head specimen and was judged useful by the surgeon.

**Conclusion:**

A hybrid robotic laser guidance system with direct visual feedback is proposed for navigated drilling and intraoperative structure localization. The system provides visual cues directly on/in the patient anatomy, reducing the standard limitations of AR visualizations like depth perception. The custom- built end-effector for the iSYS robot is transparent to using surgical microscopes and compatible with magnetic tracking. The cadaver experiment showed that guidance was accurate and that the end-effector is unobtrusive. This laser guidance has potential to aid the surgeon in finding the optimal mastoidectomy trajectory in more difficult interventions.

## Introduction

Navigated surgery in the lateral skull base is technologically and surgically challenging due to the complexity and smallness of surgical targets of the temporal bone (like the round window of the cochlea). Surgical microscopes are standardly used in ENT (Ear, Nose and Throat) surgeries, and navigated surgical stereo microscopes are rare [1], due to tedious setups and rather large impact on the surgical workflow. Furthermore, clinical acceptance is limited by the need of additional staff to run the navigation equipment. On top, precision optical tracking frequently suffers from obstructed lines of sight between camera and tracked rigid bodies [2].

Our own 25+ years of experience shows that standard pointer and monitor navigation in the lateral skull base is more desirable than a navigated microscope. An additional display for navigation poses a major mental overhead for the surgeon. Navigating with magnetic tracking might be compatible with surgical stereo microscopes [3] without an extra monitor. Moreover, experience has pointed out the need for a minimal user interface showing only the pertinent information directly in the intraoperative scene. We present a system for visualizing information to the surgeon intraoperatively without a monitor and without dedicated pointing tools.

Such a system can provide minimal information with maximum relevance to the surgeon directly in the anatomy: access path, hit / miss of the target. Similar approaches are known, the ARSys Tricorder [4], or the Probaris system [5], none of which have found widespread use in daily surgical routine, presumably due to the additional constraints introduced to surgical workflow (e.g., eye calibration or wearing AR glasses intraoperatively). When considering attention shift [6, 7] and depth-perception [8], Spatial AR systems have an advantage over conventional displays or see-through AR systems [9–11]. More recently, microscope and instrument-mounted projectors have been used to provide spatial augmented reality guidance with image projection [12–14], or instrument-mounted displays [15] to ease instrument alignment.

Our approach (“CIGuide”, Cochlear Implant Guide) projects a laser beam aligned to the surgical access path and the target in the anatomy as visual cues directly in the surgical area [16–18]. Neither extra workflow constraints nor mental loads are placed on the surgeon, giving surgically acceptable accuracy with magnetic tracking.

## Methods

In this section, we describe the hardware and software components of the prototype guidance system, the proposed workflow and the CIGuide implementation.

### Components

**Fig. 1:**
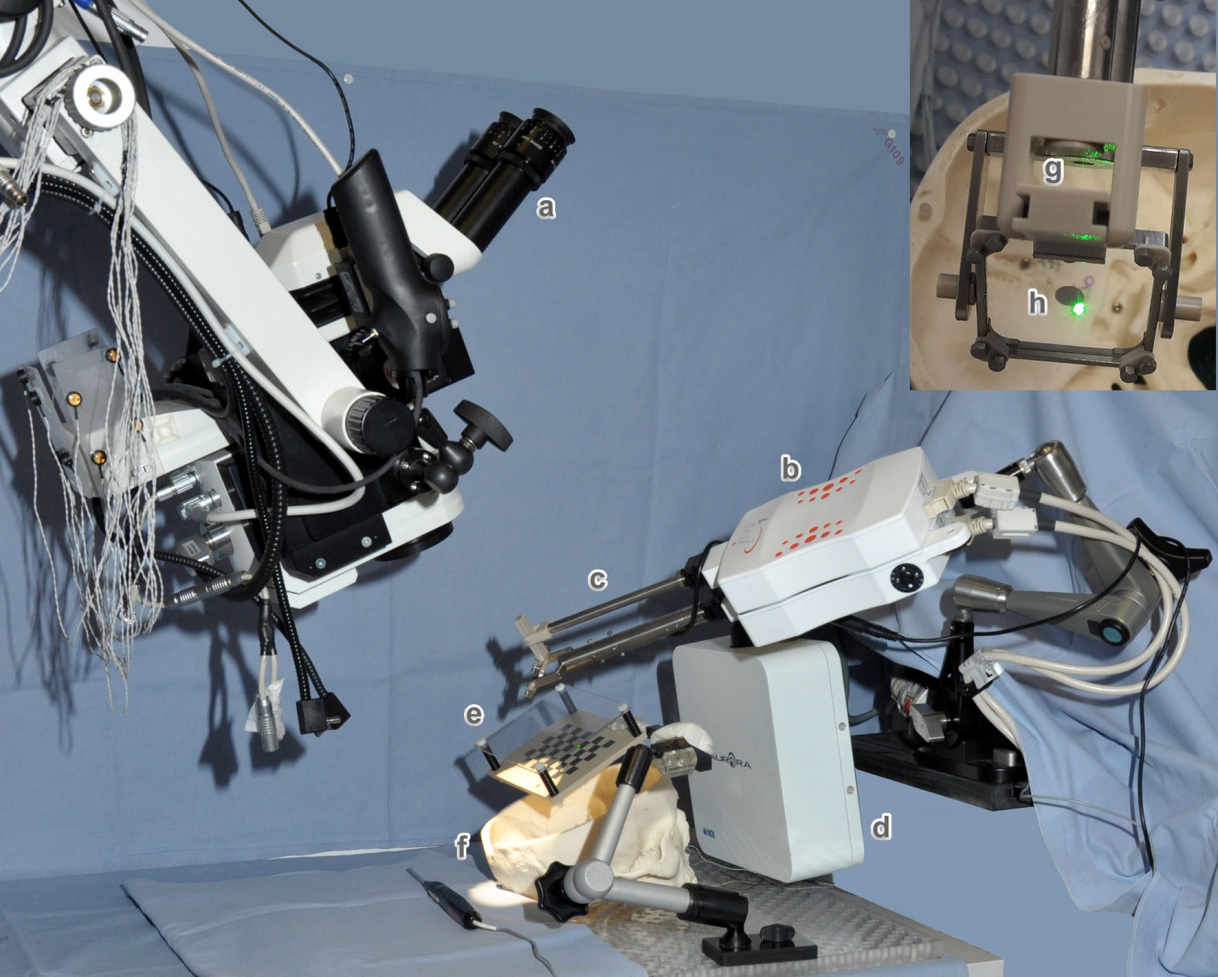
Components of the prototype setup: **a** Leica M500N stereo microscope with HD cameras, **b** iSYS-1 robot platform, **c** custom-built end-effector (medical grade-1 titanium and PEEK) housing a green laser in the lower shaft, **d** NDI Aurora field generator, **e** acrylic glass/PEEK dual plate for laser tracker co-alignment, **f** plastic skull with implanted target screws and Rhinospider sensors. The top right image shows the view a surgeon would see: **g** lower shaft with the exit pupil of laser, **h** BK7 glass with gold-coated center for deflecting the laser onto the patient

#### iSYS-1 robot

The system (Fig. 1) is a modified iSYS-1 robot [19], originally certified for needle insertion guidance with real-time fluoroscopic closed loop feedback [20].

#### Custom end-effector

**Fig. 2:**
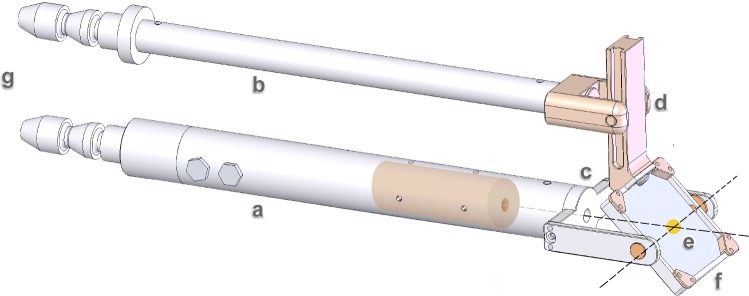
Custom-built end-effector (with length, height and width of 160 mm, $60 \,\hbox {mm} \times 40\, \,\hbox {mm}$, respectively): **a** lower shaft housing a laser (cylindrical structure), **b** upper shaft, **c** mirror holder with circular exit pupil, **d** sliding mechanism from polyether ether ketone (PEEK) converting translational upper shaft movements into mirror rotation (rotation axes are shown as dashed lines crossing the mirror center). **e** BK7 glass mirror with gold-coated center (orange dot), **f** outer mounting frame, **g** mounting to the iSYS-1 robot base. a, b, c and f are from medical grade titanium

For laser guidance while using a surgical microscope, a custom-bulit end-effector (Fig. 2, (ACMIT GmbH, Wiener Neustadt, built by Sistro GmbH, Hall i. T., both in Austria) replaces the standard needle guide extension (NGE) of the iSYS-1 robot.

It features robust laser beam guidance positioning and orienting, has minimal magnetic field distortion, (medical grade-1 titanium) and does not affect the microscope’s field of view.

The lower shaft houses a laser emitter (collimated to a 1 mm spot at 15-cm distance, 532 nm, Roithner LaserTechnik GmbH, Austria) and a $30 \,\hbox {mm} \times 30 \,\hbox {mm}$ borosilicate glass (BK7, S/D 40/20, Dr. Sztatecsny GmbH, Korneuburg, Austria) with a 3-mm-radius circular central gold coating.

The end-effector is designed for sterilization. In a real surgical setting, all but the transparent mirror can be covered in a sterile sleeve to maintain sterility.

#### Patient registration with Rhinospider

**Fig. 3:**
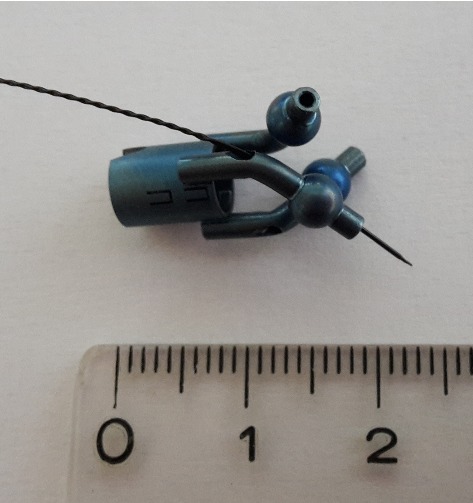
Rhinospider device, 3-ball version (titanium), with one 5D sensor inserted. The functional type would have a 5D sensor in each ball structure. The sensor protrudes the ball for better visibility

A custom-built sensor assembly (“Rhinospider”, [21]) excludes user errors during rigid body patient-to-tracker registration. The four sensors (5D, $8 \,\hbox {mm} \times 0.5 \,\hbox {mm}$, cylindrical) are isocentrically mounted in titanium spheres ($\oslash$ 4 mm) (Fig. 3) and are magnetically tracked (Aurora, Northern Digital Inc., Canada). The asymmetric four sensor/ball combinations allow unique registration. A 6D sensor is used as dynamic reference frame (DRF).

Rhinospider is inserted into the nasopharynx prior to preoperative imaging and serves as a fiducial set both for imaging and magnetic tracking and stays in place until the end of surgery. Its design allows both automated localization in CT images and during tracking. A fully automated workflow eliminates user errors and allows high-accuracy registrations potentially with submillimetric target errors.

#### Dual plate

**Fig. 4:**
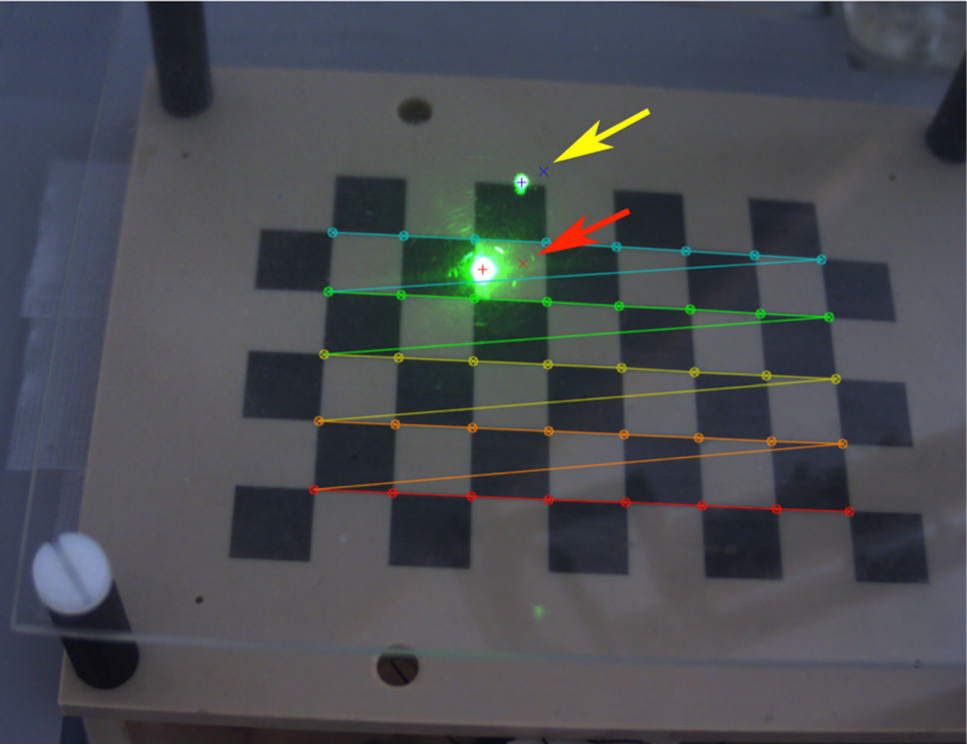
Snapshot of the AR view of the camera observing the dual plate (Dimensions: $130 \times 100 \,\hbox {mm} \times 2 \,\hbox {mm}$ plates with 25 mm separation). Upper and lower plates, checkerboard pattern, detected corners and their ordering (colored lines) are shown. The blue and red crosses show the intersections of the planned location of the guidance beam with upper and lower plates, respectively (yellow arrow and a red arrow, respectively). The green spots are the reflections of the laser beam on the plates after successful alignment. Blue and red plus signs show the detected centers of the reflections. The figure is best viewed in the digital version

The dual plate controller (DPC) aligns the guidance laser with the planned optimal access trajectory in the preoperative CT imagery without affecting the magnetic field. A two-step hybrid magnetic-optical control scheme targeting starts with EM tracking, followed by optical augmented reality tracking in the microscope’s camera view. The DPC components (Fig. 4) enable laser tracking by observing its reflections. It is built from a transparent acrylic glass ($100 \,\hbox {mm} \times 130 \,\hbox {mm} \times 2 \,\hbox {mm}$) upper plate and an opaque lower plate from PEEK ($100 \,\hbox {mm} \times 130 \,\hbox {mm} \times 5 \,\hbox {mm}$) engraved with a 10 x 6 checkerboard pattern ($10 \,\hbox {mm} \times 10 \,\hbox {mm}$ square size, Lex Feinmechanik GmbH, Grabenstätt, Germany), forming an optical reference frame. The reflections on the plates uniquely determine the laser axis in the tracker volume, allowing optimal alignment with the preoperatively planned axis

**Fig. 5:**
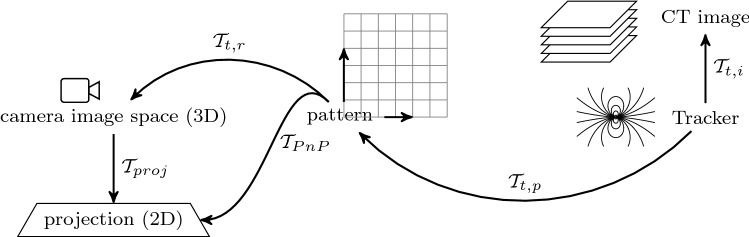
Visualization of the transformation chains used in CIGuide. For details, see the text

Four 5D Aurora sensors at well-defined asymmetric positions relative to the checkerboard pattern form a second reference frame. Both can be registered uniquely ($\mathcal {T}_{t,p}$), (see Fig. 5). The checkerboard establishes the 3D to 2D projection between pattern coordinate system and camera view $\mathcal {T}_{PnP}$, a 3D to 2D homography between pattern reference and image frames [22] which can then be decomposed into translation, rotation and projection.

$$\begin{aligned} \mathcal {T}_{PnP} = \mathcal {T}_\mathrm{proj} \circ \mathcal {T}_{t,r}, \end{aligned}$$

where $\mathcal {T}_\mathrm{proj}$ is the camera projection transform (including nonlinear distortions) and $\mathcal {T}_{t,r}$ is the rigid body transformation between the pattern and camera space [23, 24].

For fixed plate and microscope positions, $\mathcal {T}_{PnP} \circ \mathcal {T}_{t,p}$ bidirectionally connects microscope view and tracker coordinate frames.

#### CIGuide software

A prototype system (CIGuide) featuring planning, intraoperative navigation and robot control was built. Preoperative planning was implemented as a Slicer [25] module, the rest as a separate set of modules [26], based on open-source libraries [27–31]. This planning module is used to transfer the surgical access path as intended by the surgeon to the intraoperative surgical scene. Entry point, target and path, respectively, define the intraoperative path to be visualized by the laser and followed by the surgeon.

**Fig. 6:**
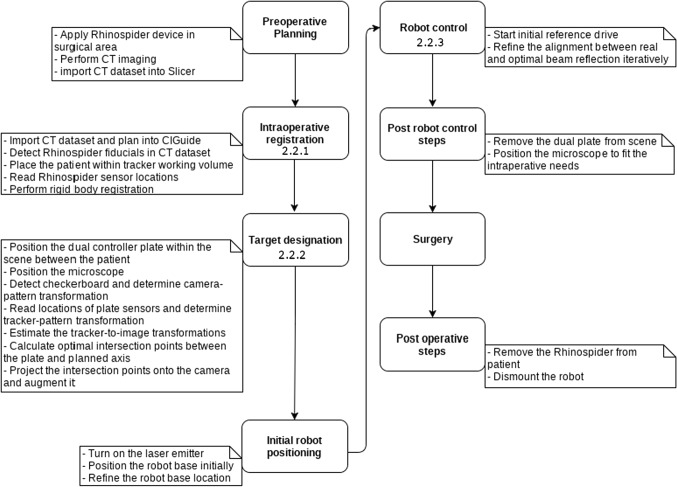
CIGuide workflow

### Workflow

The most important steps of the workflow are shown in (Fig. 6).

#### Intraoperative registration with Rhinospider

Intraoperative registration [32] between patient (Rhinospider) and patient’s radiological data requires corresponding pairs of image fiducials and tracker sensors. Rhinospider sensor balls in CT data are detected automatically with a GPU- accelerated (OpenCL, ITK) method [18]. Fifty temporally consistent sensor locations readings are averaged, while the patient maintains a fixed position relative to the tracker. Sensors and fiducials are paired by finding the permutations with minimum fiducial registration error [33]. This registration workflow with standard radiological CT imagery (0.75-mm slice thickness) can reach submillimetric application accuracy in regions close to the sensors such as the cerebello-pontine angle or the lateral skull base [21].

#### Target designation

At the “Initial Robot Positioning” step, the operator positions the iSYS-1 robot base within the scene and fixes it to the operating table. The laser (viz., the end-effector) is manually positioned at the planned position by directly observing the guidance beam on the patient. The robot has a limited working volume and should have a roughly optimal pose to successfully reach the planned trajectory during robot control. Once fixed, a few reference movements suffice to decide whether the target location is reachable or not. If not, the robot position needs to be improved.

Next, the DPC is placed on the patient and target designation starts: As an aid, a live AR view shows the calculated reflections on both plates where the guidance laser should hit both plates (Fig. 4).

1. Transform preoperatively planned target axis into pattern space, $\mathcal {T}_{t,p} \circ \mathcal {T}_{t,i}^{-1}$.
2. Calculate the intersections of planned axis with the two plates, $t_\mathrm{lp}$ and $t_\mathrm{up}$.
3. Project intersection points ($t_\mathrm{lp}$, $t_\mathrm{up}$) in the live camera image.
4. Position end-effector such that the laser hits the projected loci on both plates.

#### Robot control

Next, a two-step iterative closed loop feedback controller based on visual feedback moves the robot to the desired location [34].

The two main steps executed by the controller are:

1. Reference Movement Step: based on visual feedback, the end-effector is moved to a few predefined locations to decide whether a target is reachable from the current position. If not, the robot must be repositioned.
2. Iterative Refinement Step: based on the difference between the observed and desired locations of the reflections, the robot reduces feedback error with a correctional move. This is iterated until the feedback error reaches a predefined threshold, or the maximum number of iterations is reached.

#### Refraction correction

Refraction on the upper plate requires correcting the measured positions on the lower plate before it can be used to determine the true location/orientation of the beam in space (Snell’s law) [18].

## Evaluation

### Experimental setup

**Fig. 7:**
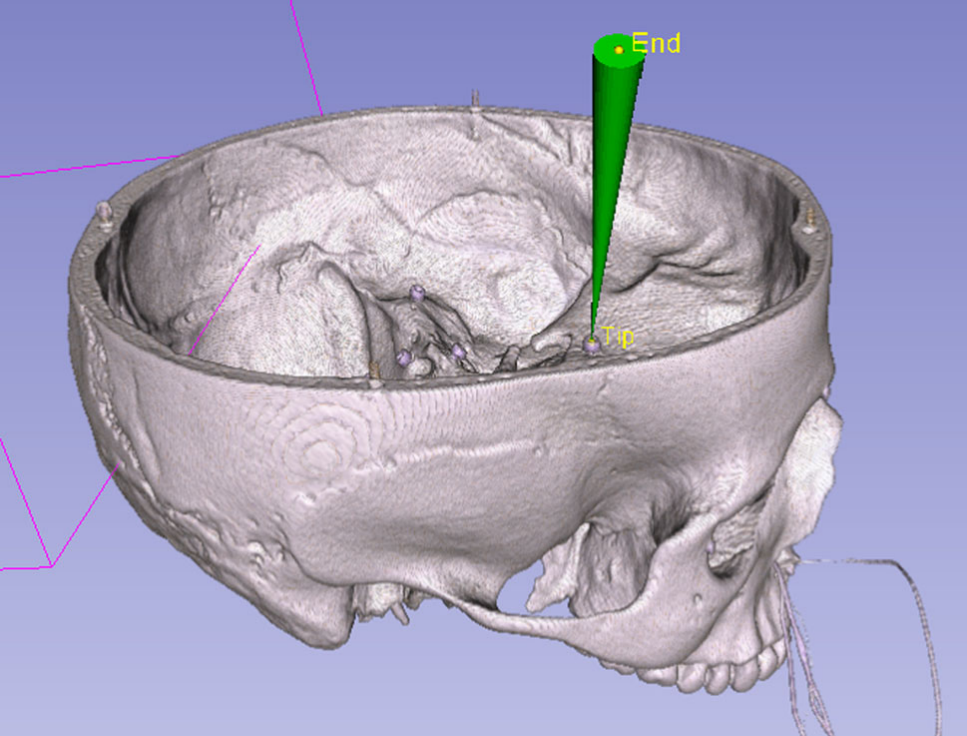
For testing purposes, a fictitious preoperative plan (cone) is visualized on top of the volume-rendered patient dataset. Yellow points show the tip and endpoints defining a cone. The tip is positioned at the anatomic target; here, on one titanium screw, the endpoint is the entrance region

**Fig. 8:**
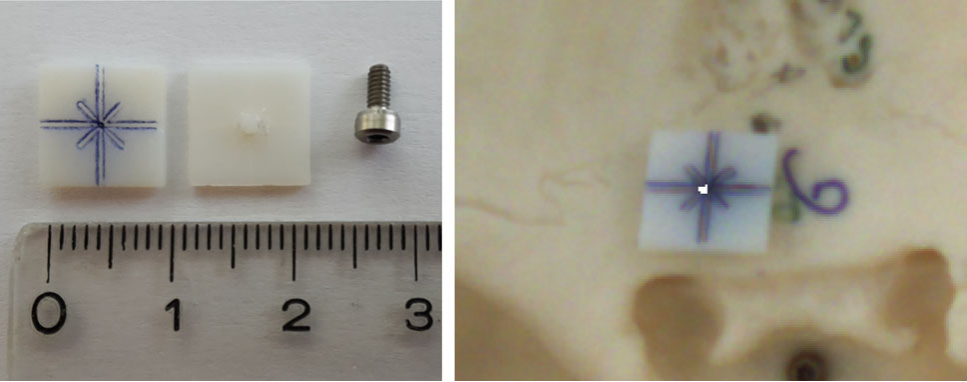
Left part: front side of the 3D-printed evaluation plate ($10 \,\hbox {mm} \times 10 \,\hbox {mm} \times 1 \,\hbox {mm}$), painted blue on the front for better contrast and rear side with the protrusion on the back side fitting the 2-mm titanium screw. Right image: combined sample image for evaluation of target S6

The accuracy of the proposed system was evaluated on a plastic skull phantom with inserted Rhinospider sensors and titanium screws at various locations. For each location, a target axis was defined in the preoperative planning module (Fig. 7). The plastic skull was then positioned randomly in the optimal working volume of the Aurora device so as to allow viewing it with the microscope. To compensate for illumination losses in the stereo microscope, an external high-resolution camera (uEye UI-1245LE, IDS GmbH, Tamron 13VM550ASII CCTV optics, Tamron Europe GmbH, both Germany) was utilized to observe the dual plate. A small, plastic target plate of $10 \,\hbox {mm} \times 10 \,\hbox {mm}$ was designed to fit the target screw head. (Fig. 8). The plate was 3D printed with a cross-shaped indentation to fit the head of the target screw. During the evaluation runs, the plate was put on top of each target to provide a reference plane.

### Evaluation procedure

The whole procedure was repeated ten times for each of the five different targets. At each iteration, two images were captured with different exposure times: one with the laser off (reference image) where the cross indentation is clearly visible, and a measurement image with a short exposure time and with the laser turned on. The measurement image was thresholded, eroded into a few pixels to show the beam’s center and then overlaid as a white pixel layer on the image with normal exposure. For each image target position, the center of the laser spot, top, bottom, left and right endpoints of the engraved cross was marked up manually and used to reconstruct the millimetric displacement error between the center of the cross and the center of the laser spot. The distance of the spot to the target was directly measured on the camera images that were calibrated and undistorted. The center and corner of the target plate and the laser spot center were manually annotated, and the millimeter distance was estimated from the pixel distance using the camera calibration.

## Predicting guidance uncertainties at the target

**Fig. 9:**
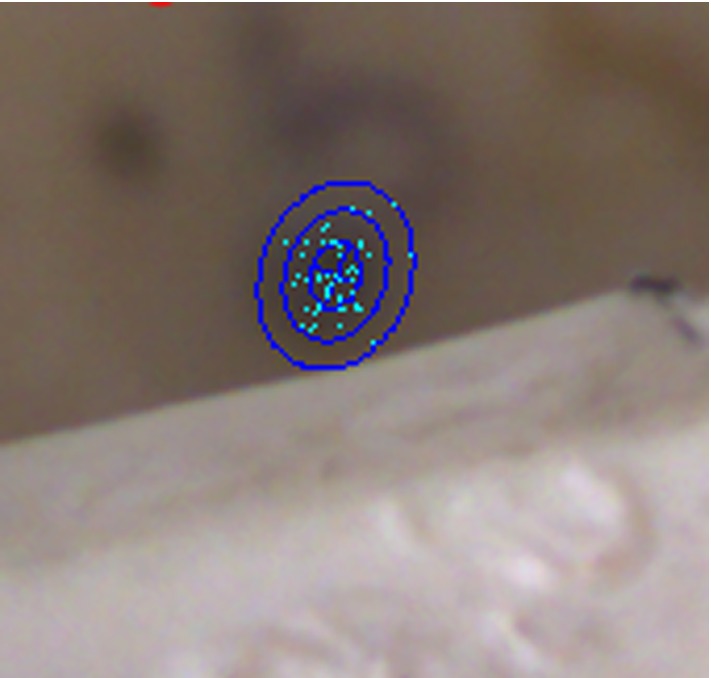
AR view with target uncertainty. The light blue points show the intersections of the simulated trajectories with the target plane. The blue ellipses show the probability iso-contours for one, two and three sigma distances from the mean location. One sigma distance was 0.876 mm and 0.726 mm along the semimajor and semiminor axes, respectively

The uncertainty of the approach at a given target including the dual plate location was estimated with a Monte Carlo simulation of probable guidance trajectories, based on [18, 33, 35]. The prediction method is also used to visualize the expected uncertainty of a given setup before the actual robot control was executed (Fig. 9). After experimental evaluation, the validity of these predictions was checked against the measurements.

## Cadaver experiment

**Fig. 10:**
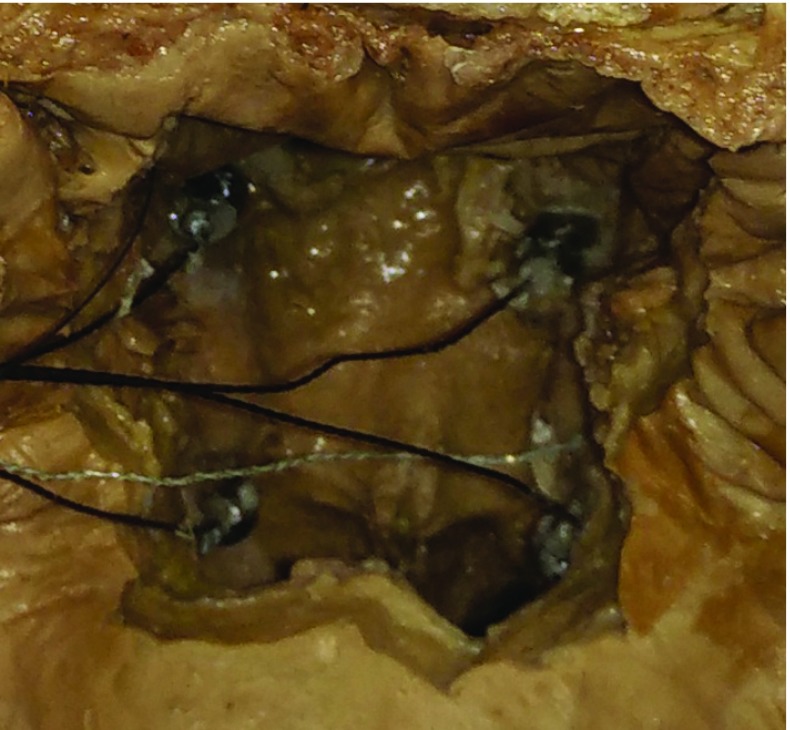
Caudal–cranial view of the cadaver with the soft palate removed. The four Rhinospider markers with the inserted and centered 5D Aurora sensors were glued to the nasal mucosal membrane

For evaluating, the guidance system was used for guidance in a cadaver mastoidectomy. First, removing the mandible and the cranial remains of the neck was from an anatomic specimen gave access to the choana from posterior. A Rhinospider assembly with a 6D DRF was fixed (superglue) to the nasal mucosa (Fig. 10). Standard clinical cranial CT images (1-mm slice thickness) were created.

During preoperative planning, the head of the incus, which can easily and precisely be identified in patient and radiological imagery, was designated as a target.

**Fig. 11:**
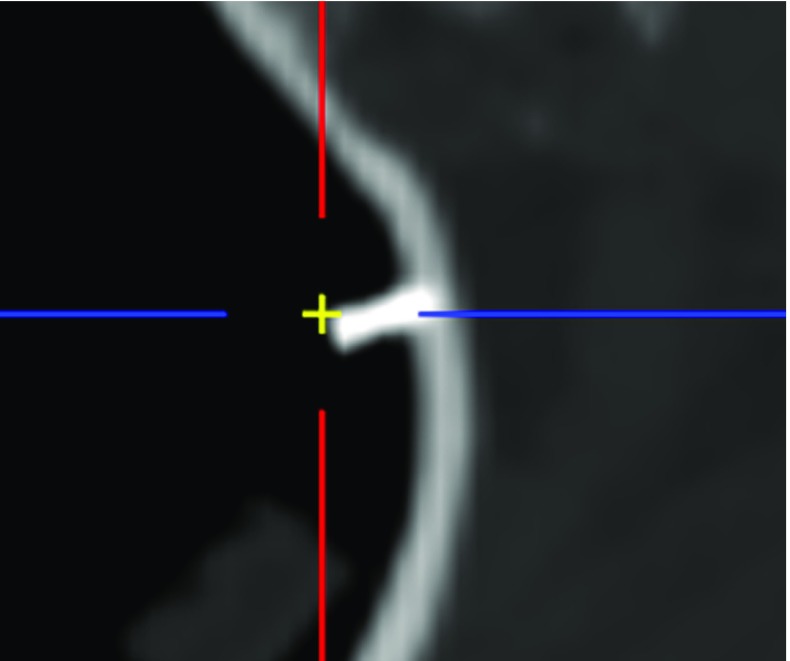
Partial screenshot of the navigation while the accuracy of the registration on the cadaver was qualitatively evaluated by localizing the implanted screws with the navigation probe

Patient registration was repeated four times with slightly different orientations relative to the magnetic field emitter. The RMS FRE error was $0.84 \pm 0.13$ mm. The registration was qualitatively validated by touching the implanted 2-mm titanium screws with the navigation system’s probe and checking the error in the CT images (Fig. 11). After successful validation of the registration, the surgeon performed the drilling steps of mastoidectomy with the microscope while the guidance beam was active.

## Results

The resulting target accuracies during the quantitative evaluation on the plastic skull are presented in Table 1. The system is able to reach an average target accuracy error of 1.2 mm ± 0.5 mm, which is close to the limit achievable with the magnetic tracking system in use.

The overall predicted target standard deviation of the target accuracy for the tested plate locations was ± 0.52 mm, which corresponds nicely to the measured experimental uncertainty.

**Fig. 12:**
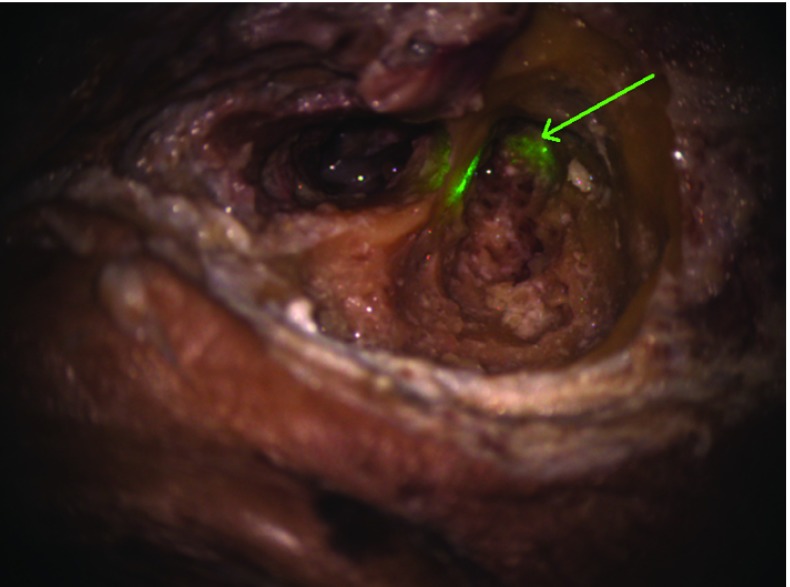
Screenshot captured from the live view of the surgeon through the microscope with the CIGuide guidance beam in place. On the top left side is the ear canal, with the malleus visible in the middle ear cavity, after moving the eardrum. To the right of the posterior wall of the outer ear canal is the mastoid cavity, with the guidance beam reflecting from the head of the incus. A partial reflection of the beam is also visible on the posterior wall. After targeting and drilling, the error of the guidance beam relative to the preoperatively planned location (marked by the green arrow) was approximated to be 2 millimeters

System setup including control iterations after patient registration added approximately 15 minutes to the intraoperative workflow. On the cadaver, the target accuracy at the incus bone was subjectively evaluated by the surgeon and the assistant after the drilling step (Fig. 12). The accuracy of the guidance beam at the end of the experiment was estimated to be 2 millimeters. Overall, the surgeon stated that the guidance was not obstructing the view during the mastoidectomy and that it can be helpful for more complicated cases.

**Table 1:** Measured target accuracies in the plastic skull experiment with targets and their standard deviations in millimeters

Target number	Error and standard deviation in mm
S1	$1.44 \pm 0.45$
S2	$1.24 \pm 0.56$
S3	$1.16 \pm 0.55$
S4	$1.19 \pm 0.51$
S5	$0.95 \pm 0.43$
$\bar{S}$	$1.2 \pm 0.5$

S1: inner ear canal, S2: eminentia arcuata, S3: horizontal course of the internal carotid artery, S4: clivus, S5: apex of the orbit. $\bar{S}$ is the mean value of the other positions

## Discussion and conclusions

It is concluded that the magnetic tracking offers an easier approach to intraoperative tracking and user error-free registration without uninterrupted line-of-sight requirements. Sensors are small enough to be positioned close to the relevant surgical structures inside the body. Rhinospider technology is similar to nasal stents (e.g., http://www.alaxo.com/alaxolito_eng.html, Alaxo, Germany) that patients very well tolerate easing nasal breathing in case of nasal congestion and for sports.

Once registration and plate position were determined, a feedback controller utilizes HD camera tracking for robotic laser beam alignment. So the robotic platform inside the magnetic field does not need to be magnetically tracked, which makes the robot design easier. Optical tracking is far more accurate and allows positioning the robot platform far off the region of interest, or even outside the working volume of the tracker.

This hybrid tracking approach enables direct tracking of the guidance laser beam, resulting in significantly better target accuracy than direct magnetic tracking of the robotic end-effector itself. Other designs of control plate and sensor mountings to it could somewhat further reduce the target error. Further phantom experiments with anatomic specimens and surgeons performing “real” interventions are planned to determine the system’s behavior under more realistic conditions.

The system shows a promising potential in our initial tests to be a laser guidance platform that is easy to use, is built from standard elements and can be utilized during surgery without major additional workload on the medical personnel. The system allows in situ visualization of information with a fairly small impact on the surgeon’s mental workload and can easily be integrated into existing operating theatres and workflows.

The CIGuide system as presented builds on Rhinospider technology applied to the nasopharynx. This is no limitation for microscopic interventions at the lateral skull base of all kinds. The insertion of the short-term (less than one day) registration device in the nasopharynx has shown promising guidance in our laboratory investigation. Endoscopic interventions at the anterior skull base, the pituitary, or beyond, are not intended as surgeries to benefit form this technology. The authors are aware of the limits of using one laser beam as a guidance aid. Preliminary work with a second laser to encode the spatial target position as an intuitive surgical visualization is under way; due to the complexity of the issue, it is foreseen to be published separately.
